# Vein depth and diameter as predictive indicators of visibility and palpability during venipuncture in healthy volunteers

**DOI:** 10.1371/journal.pone.0323367

**Published:** 2025-05-09

**Authors:** Miharu Matsumoto, Ayami Tateishi, Hiromitsu Kobayashi, Nobuko Hashiguchi

**Affiliations:** 1 Department of Health Sciences, Kyushu University, Fukuoka, Japan; 2 Department of Health Sciences, Graduate school of Medical sciences, Kyushu University, Fukuoka, Japan; 3 Ishikawa Prefectural Nursing University, Kahoku, Ishikawa, Japan; Shaanxi Provincial People's Hospital, CHINA

## Abstract

**Background:**

The difficulty of venipuncture depends on the visibility and palpability of the vein and is particularly challenging in older adults, patients with abnormal body mass index, and those with chronic diseases. In these populations, vessel fibrosis, scarring, skin atrophy, and subcutaneous fat reduce vein visibility and palpability, making venous access more challenging. Previous studies have shown that visibility and palpability are associated with vein depth and diameter, respectively. The current study therefore aimed to evaluate the validity of vein depth and vein diameter as predictive indicators of vein visibility and palpability during venipuncture.

**Methods:**

Three nurses evaluated the visibility and palpability of superficial veins suitable for venipuncture in 96 healthy volunteers who participated in this study. Using ultrasound imaging devices, the depth and diameter of superficial veins were measured, after which receiver operating characteristic (ROC) curves were created to identify the optimal cutoff values for predicting vein visibility and palpability.

**Results:**

Both vein depth and diameter were found to be independent predictors of visibility and palpability (p < 0.05). ROC analysis showed that vein depth was a significant predictor of visibility (AUC = 0.901, 95% CI = 0.834–0.967) and palpability (AUC = 0.724, 95% CI = 0.619–0.829). The optimal cutoff values for vein depth were 1.89 mm for visibility discrimination, with 28.1% of veins exceeding this threshold, and 1.44 mm for palpability, with 59.4% of veins exceeding this threshold. On the other hand, vein diameter was less accurate than vein depth in predicting visibility and palpability (AUC < 0.7).

**Conclusions:**

Vein depth is an effective predictor of vein visibility and vein palpability. This finding is expected to inform the development of assistive technology for visual and palpatory examinations.

## Introduction

Peripheral venipuncture, which includes blood sampling and catheter placement, is a basic medical procedure utilized for diagnosis, treatment, and evaluation of treatment efficacy. Despite the considerable invasiveness of this procedure, which can manifest as pain and potential complications after the puncture, initial success rates have range from approximately 65%–90% [1–5].

Repeated punctures due to venipuncture failure not only cause the formation of a hematoma and make re-puncture difficult but also promote long-term damage to the vessel wall. Failed venipuncture has been associated with difficult intravenous access (DIVA) [6], which delays diagnosis and treatment initiation [7]. Moreover, DIVA increases the likelihood of waiting over 240 min for treatment by a factor of 5.01 compared to non-DIVA [8]. This issue is a particularly important in outpatient settings where wait times decrease patient satisfaction [9].

DIVA depends on the visibility and palpability of the veins, and venipuncture is particularly challenging in older adults, patients with abnormal body mass index, and those with chronic conditions [10,11]. In these patient groups, vascular fibrosis, scarring, skin atrophy, and subcutaneous fat reduce venous visibility and palpability, making venous access more challenging.

To address this issue, several scales have been recently developed to identify cases with DIVA and facilitate the early implementation of assisted puncture techniques [1,2,4,12,13], including ultrasonography guidance or vein visualization devices [14,15]. Notably, these scales assess vein visibility and palpability, two factors closely associated with DIVA, serving as critical predictive indicators for evaluation purposes [1,2,4,12,13]. As venous visibility and palpability can be easily assessed through visual and palpatory examinations by a venipuncture clinician, they offer a convenient means of DIVA prediction. However, these assessments are inherently subjective and dependent on the clinician’s experience, thereby decreasing their objectivity.

Advances in vein visualization technology using ultrasonography imaging systems have facilitated the quantitative evaluation of visibility and palpability, which were previously based on subjective clinician assessment [16–18]. Previous studies have demonstrated that vein depth and cross-sectional area influence visibility [16], with superficial veins deeper than 2.3 mm before tourniquet application being less visible [17]. Another study showed that palpability following tourniquet application was negatively correlated with vein depth but not with vein diameter [18].

However, previous studies have used different measurement conditions, such as the presence or absence of a tourniquet. In addition, studies examining the relationship between palpability and vein depth and diameter are particularly limited; hence, the extent of their influence remains unknown. Therefore, comparing and integrating factors affecting visibility and palpability has been difficult given the limited findings in previous studies.

Assuming that ultrasound imaging could predict the visibility and palpability of veins, the technique could be expectedly utilized an assistive technology for visual and palpatory examinations. Furthermore, when combined with recently developed portable vein finders [19], and AI-based automated measurement techniques [20,21], this technology could be expectedly implemented for screening cases with venipuncture difficulties.

The current study aimed to evaluate the validity of vein depth and diameter as predictors of visibility and palpability of peripheral superficial veins for venipuncture.

## Materials and methods

### Participants

The participants in this cross-sectional observational study were recruited from April 22 to June 20, 2024 through posters on the university bulletin board and announcements on the university website. The measurement period was from May 8 to June 25, 2024.

This study employed a nonprobability convenience sampling method, wherein participants who spontaneously responded were selected on a first-come, first-served basis and screened for inclusion and exclusion criteria. The inclusion criteria encompassed participants aged ≥18 years and experienced blood collection. The exclusion criteria were those being treated for any disease, pregnant, or potentially pregnant. The reference population for this study comprised healthy adults who have had general experience of blood collection.

The sample size was determined based on a power analysis using G*Power for two independent groups. Based on a previous study [17], the effect size for the difference in venous depth between the visible and invisible veins was approximately d = 0.96. A sample size of 48 participants was demonstrated to provide 80% statistical power at a 0.05 alpha level. A previous study with a similar methodology [18] was also considered to determine the sample size.

A total of 96 healthy volunteers participated in this study (15 males and 81 females). The final sample had a mean age of 20.9 ± 2.2 years (23.0 ± 3.7 and 20.5 ± 1.6 years for males and females, respectively), mean height of 160.2 ± 7.2 cm (169.4 ± 4.3 and 158.4 ± 6.2 cm for males and females, respectively), and mean body weight of 54.0 ± 9.2 kg (64.0 ± 10.9 and 52.2 ± 7.6 kg for males and females, respectively).

This study was conducted in accordance with the ethical principles of the Declaration of Helsinki and received ethical approval from the Ethics Committee for Clinical Research of Kyushu University Hospital (approval number 24005–00). Participants received oral and written explanations regarding the study and provided their written consent to participate in this study.

### Procedures

The measurement environment was as follows: room temperature, 24.5 ± 0.7 °C; humidity, 62.5 ± 11.3% RH; and illuminance, 566.5 ± 17.71x. Participants sat on a chair and self-reported the side of the arm that was frequently selected in previous blood sampling. If the participants did not select a specific side, the non-dominant side was selected. The upper extremities were placed on an 82-cm-high table with their palms facing up and their forearms and upper arms being secured using belts.

After resting for at least 1 min, the evaluators performed visual and palpatory examinations. Superficial veins suitable for blood sampling were selected and evaluated for visibility and palpability. The evaluators for this process were three nurses with at least 5 years of clinical experience. Only the first evaluator decided on the target vein, with the other two evaluators evaluating the vein selected by the first evaluator (the marked site). Visible and palpable in this study were defined as follows: “visible” veins were defined as those that were discernible without tourniquet application based on their difference from the surrounding skin color, whereas “palpable” veins were defined as those that were palpable and elastic. Each evaluator was allowed an evaluation time of 15 s, with the order of ratings randomly assigned. In this study, veins were considered visible or palpable after two or more evaluators agreed on the evaluations.

Thereafter, ultrasound imaging of the vein at the marked site was performed. A warmed gel coating at least 1-cm thick was applied over the skin to prevent the vein from collapsing under pressure from the probe. Using a 10-MHz linear probe (Mirco, Sigmax Co, Tokyo, Japan), a B-mode transverse still image was captured and recorded on the device. The ultrasound imaging system was operated by one researcher who received adequate training and had experience operating similar systems in previous studies. Images were captured uniformly 60 s after the end of the examination. The three evaluators assessing vein visibility and palpability and the researcher capturing ultrasonographic images were blinded to each other’s assessment and imaging results, ensuring that all evaluations and image acquisitions were independently conducted.

### Image analysis

Images recorded on the ultrasound imaging system were converted to JPEG files and imported into ImageJ software [22]. Vein depth and diameter were analyzed using the same procedure described in previous studies [23]. All images were analyzed blindly and randomly by one researcher.

### Statistical analysis

Vein depth and diameter were compared according to their relationship with visibility and palpability, respectively, using the Mann-Whitney U test. Furthermore, the difficulty level of venipuncture was defined as “easy” for visible and palpable, “moderate” for visible or palpable, and “difficult” for invisible and impalpable, based on previous studies [1,2,4,12,13]. The Kruskal-Wallis test was employed to compare the median values across the three independent groups. When a significant difference was observed, post-hoc analysis was performed using Dunn’s test with Bonferroni correction to identify specific group differences.

Factors significantly predicting visibility and palpability were determined using multivariate regression analysis, with both vein depth and diameter being included in the model as predictors. Predictive accuracy was evaluated by mapping the receiver operating characteristic curves (ROC) and calculating the area under the curve (AUC). Cutoff values for vein depth and diameter were determined based on the Youden Index. All statistical analysis were conducted using R version 4.2.2 for Windows, with a p value of < 0.05 indicating statistical significance.

## Results

Among the included participants, 22 (22.9%) and 74 (77.1%) selected their right and left upper extremity, respectively. The evaluator selected the median cubital vein in 49 cases (51.0%), the cephalic vein in 36 cases (37.5%), and the basilic vein in 11 cases (11.5%). The mean vein depths for the median cubital vein, basilic vein, and cephalic vein were 1.91 ± 1.02 mm, 1.61 ± 0.71 mm, and 1.54 ± 0.54 mm, respectively. Vein diameter was largest in the median cubital vein (3.51 ± 1.13 mm), followed by the cephalic vein (2.88 ± 0.81 mm) and basilic vein (2.82 ± 0.60 mm).

Table 1 compared vein depth and diameter in the presence and absence of visibility and palpability, respectively. Visible veins were significantly deeper than were invisible vein (p < 0.001). Palpable veins were significantly larger than were impalpable veins in both depth and diameter (p < 0.001 and p = 0.013, respectively).

**Table 1:** Comparison of vein depth and diameter according to visibility and palpability.

		n	Depth (mm)	p-value ^a^	Diameter (mm)	p-value ^a^
Visibility	visible	85	1.50 (1.20–1.76)	<0.001	2.96 (2.54–3.65)	0.164
	invisible	11	2.26 (2.19–2.62)		3.47 (2.63–4.93)	
Palpability	palpable	25	1.26 (1.19–1.52)	<0.001	3.47 (2.96–4.06)	0.013
	impalpable	71	1.64 (1.37–2.18)		2.92 (2.41–3.48)	

Results are presented as median (IQR). Visible veins were deeper than invisible veins. Palpable veins were also significantly deeper than impalpable veins and had significantly larger vein diameters than impalpable veins.

a p-values are calculated using the Mann–Whitney U test.

**Fig 1:**
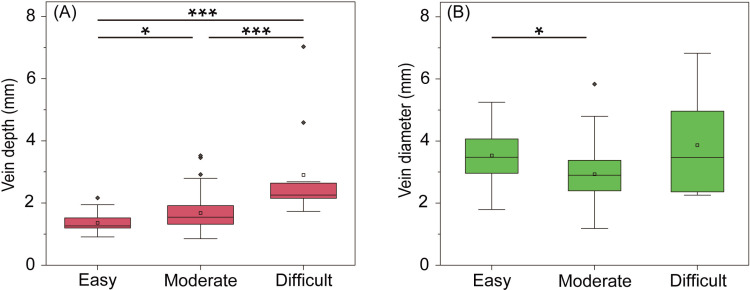
Comparison of (A) vein depth and (B) vein diameter between difficulty groups. The center line of the box represents the median, whereas either end of the box represents the first quartile (Q1) and the third quartile (Q3). The whiskers extending from the box indicate the range of the data (1.5 times from Q1 and Q3, respectively), and the black diamonds outside the box are outliers. Additionally, the white square indicates the mean. p-values are marked in the figure: *** p < 0.001, * p < 0.05.

A comparison of vein depth and diameter according to venipuncture difficulty is demonstrated in Fig 1. Accordingly, 25, 60, and 11 participants were classified into the difficult, moderate, and easy groups, respectively. The median (interquartile range) vein depths for the difficult, moderate, and easy groups were 2.26 (2.19–2.62), 1.55 (1.32–1.88), and 1.26 (1.19–1.52) mm, respectively. The corresponding vein diameters were 3.47 (2.63–4.93), 2.90 (2.42–3.36), and 3.47 (2.96–4.06) mm, respectively. Vein depth differed significantly between the three difficulty groups (p < 0.001). The post-hoc Dunn test with Bonferroni correction revealed significant differences between all group’s pairs (difficult vs. moderate: p < 0.001; difficult vs. easy: p < 0.001; and moderate vs. easy: p = 0.031). Similarly, significant differences in vein diameter were observed between the three difficulty groups (p = 0.006), with paired group comparisons showing significant differences only for the moderate and easy groups (p = 0.011).

Multivariate analysis revealed that both vein depth and diameter were independent predictors of reduced visibility and palpability (Table 2).

**Table 2:** Multivariate regression analysis to explain the decrease in visibility and palpability.

Visibility		Odds Ratio	95% Confidence Interval	p-value	VIF
	Depth (mm)	9.66	2.53–36.8	<0.001	1.20
	Diameter (mm)	2.15	1.09–4.26	0.028	1.20
**Palpability**		**Odds Ratio**	**95% Confidence Interval**	**p-value**	**VIF**
	Depth (mm)	7.46	1.87–29.8	0.001	1.01
	Diameter (mm)	0.60	0.36–0.98	0.043	1.01

**Fig 2:**
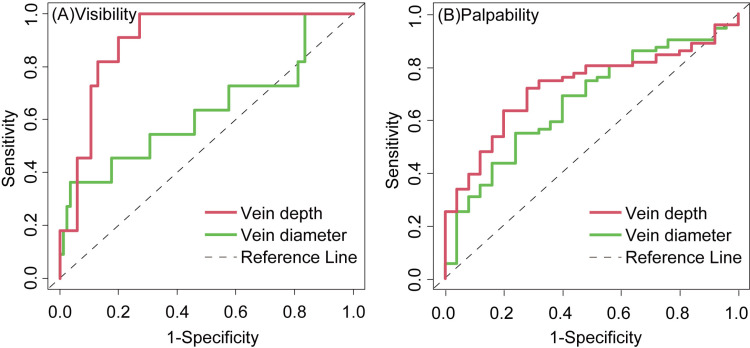
Receiver operating characteristic curves for predicting (A) visibility and (B) palpability. Lines indicate vein depth (red) and vein diameter (green). The dotted line indicates the reference line.

ROC curves for vein depth and diameter as discriminators of visibility and palpability were created (Fig 2). Vein depth was a strong predictor of visibility, with an AUC of 0.901 (95% CI = 0.834–0.967), while vein diameter showed a lower predictive ability, with an AUC of 0.630 (95% CI = 0.419–0.841).For palpability, vein depth had a moderate predictive ability (AUC = 0.724, 95% CI = 0.619–0.829), and vein diameter had a slightly lower predictive ability (AUC = 0.668, 95% CI = 0.548–0.787). This finding indicates that vein depth is superior to vein diameter as a predictor of both vein visibility and palpability.

The cutoff value for vein depth identifying visibility was 1.89 mm (sensitivity, 100%; specificity, 72.9%), with 28.1% of the veins exceeding this threshold. For palpability, the optimal cutoff value for vein depth was 1.44 mm (sensitivity, 71.8%; specificity, 72.0%), with 59.4% of the veins exceeding the threshold.

## Discussion

Venipuncture of invisible and impalpable veins is quite challenging, leading to reduced first-time success rates for such veins. Therefore, clinicians who perform venipuncture are required to perform a thorough visual and palpatory examination prior to the procedure to ensure safe puncture. The current study evaluated the visibility and palpability of veins considered suitable for venipuncture and measured their depth and diameter.

### Effectiveness of vein depth and diameter as predictors of visibility and palpability

Studies have shown that the visibility and invisibility of veins in the upper extremity can be distinguished at a vein depth of 2.3 mm [17]. However, the cutoff value for vein depth in the current study was 1.89 mm, which was shallower than that reported in previous studies. The difference between these two studies is that our study selected veins that were intended for blood sampling. In other words, our findings suggest that for the purpose of blood sampling, selecting a vein located shallower than the conventional criteria for a visible vein will more efficiently select the target vein for puncture.

In the current study, palpability was predicted according to vein depth, at a cutoff value of 1.44 mm. Vein depth is the distance from the skin surface to the superior wall of the vein, between which anatomic subcutaneous fat is present. Prior studies have shown that vein depth was positively correlated with fat mass [23]. The results of the current study suggest that palpability decreases with increasing vein depth, which is also associated with increased subcutaneous fat mass.

Aging and mechanical stimuli (e.g., repeated needle punctures, administration of anticancer drugs) alter the structure of the venous wall [24]. Moreover, dehydration can decrease intravascular pressure and collapse the vein diameter [25]. These factors contribute to a possible decrease in vein palpability. However, given that most of the participants included herein were healthy volunteers in their 20s, structural alterations in the walls of the veins or vessels collapse is highly unlikely. The aforementioned findings suggest that the physical distance from the skin surface to the vein, especially the amount of subcutaneous fat mass, can mainly influence the decrease in palpability.

On the other hand, our findings showed that vein diameter was not a predictor of visibility or palpability. This result is similar to that reported in previous studies, which found that vein cross-sectional area was not a relevant factor of visibility [17,18]. Hence, our findings suggest that simply having a large vein diameter does not lessen the difficultly of detection on visual and palpatory examination. Based on this finding, future studies are required to consider the flexibility of the tissue surrounding the vein and physiologic factors (e.g., blood flow and flexibility of the vein wall).

### Predicting the difficulty of venipuncture

A novel finding of the current study is the validity of vein depth as a predictor of both visibility and palpability. In the present study, the difficulty of venipuncture was stratified into “easy,” “moderate,” and “difficult” based on visibility and palpability, which are requirements for determining DIVA in previous studies [1,2,4,12,13]. Our results showed that vein depth was deepest in the difficult group, followed by the moderate and then easy groups (Fig 1). However, this trend was not observed for vein diameters. This finding suggests that vein depth is an important objective indicator of the difficulty of venipuncture.

Our results showed that palpability decreased as vein depth increased deeper than 1.44 mm, with visibility also decreasing as depth exceeded 1.89 mm. Interpreting these results in the context of venipuncture difficulty reveals that at vein depths shallower than 1.44 mm before tourniquet application, the vein is visible and palpable, making venipuncture easy. On the other hand, at vein depths between 1.44 and 1.89 mm, the vein is visible but impalpable, making venipuncture moderately difficult. Therefore, more careful puncture positioning is recommended for veins in this range. Furthermore, at vein depths deeper than 1.89 mm, the vein is invisible and impalpable, making venipuncture more difficult. In such circumstances, puncture by a skilled clinician and the use of assistive technologies (e.g., ultrasound guides and vein finders) can be considered.

### Limitations

The current study has several limitations that warrant discussion. First, this study was limited to healthy volunteers, most whom were young females. Thus, bias in subcutaneous fat mass and venous vessel wall structure, which can potentially affect vein visibility and palpability, could be present. Therefore, further validation in a larger cohort with more participants is required. Second, vein depth and diameter were the only predictive indices included in the current study. Furthermore, vein diameter showed low predictive accuracy for both visibility and palpability (AUC < 0.7). Therefore, future studies need to improve the prediction accuracy, including the addition of other indices (e.g., blood flow and venous compliance).

## Conclusion

The current study showed that vein depth was an effective predictor of vein visibility and palpability before tourniquet application. Both visibility and palpability increased at vein depths shallower than 1.44 mm but decreased at vein depths deeper than 1.89 mm. These findings offer valuable insights into the anatomical factors affecting venipuncture success. Integrating vein anatomy evaluation into existing portable vein finders and AI-based automated measurement technologies may significantly affect the development of assistive technologies for visual and palpatory examinations. Furthermore, to investigate the effects of physiological conditions on vein visibility and palpability, the study population should be expanded to encompass patient groups in future studies. This future direction would enable the development of a comprehensive and clinically adaptable predictive model of venipuncture success.

## Supporting information

S1 File Raw data. (XLSX)

S1 Checklist PLOSOne_Human_Subjects_Research_Checklist. (DOCX)
